# Rapid geographic expansion of local dengue community transmission in Peru

**DOI:** 10.1371/journal.pntd.0013001

**Published:** 2025-04-17

**Authors:** Edson J. Ascencio, Paloma M. Carcamo, Gabriel Carrasco-Escobar

**Affiliations:** 1 Health Innovation Laboratory, Institute of Tropical Medicine Alexander von Humboldt, Universidad Peruana Cayetano Heredia, Lima, Peru; 2 Yale School of Public Health, Yale University, New Haven, Connecticut, United States of America; Oregon Health and Science University, UNITED STATES OF AMERICA

Dengue is currently the most significant vector-borne disease in Peru, with its incidence increasing markedly over the past few years. In particular, 2023 saw a substantial rise, with 251,605 confirmed cases reported, making it the highest incidence recorded in the country’s history [1]. Although cases were reported in Peru during the 1950s, the epidemic in 1990 was the first laboratory confirmation of dengue indigenous transmission in Peru [2]. Confirmed cases in 2023 were approximately 9-fold the average number during the previous 5 years (29,841 confirmed cases) and 4.2-fold the number during 2017 (59,303 confirmed cases), the year of the largest previous national dengue outbreak [3]. We hypothesized that, in Peru, the apparent confluence of urbanization, climate change, and increased human migration has led to the worst dengue epidemic in the country’s history, peaking in recent years. In this comment, we aimed to describe the rapid geographic expansion of local community transmission of dengue in distinct natural regions of Peru over the past two decades, particularly toward districts that have historically been free of dengue transmission.

We used the reported probable and confirmed dengue cases from 2000 to 2023 (Peruvian Ministry of Health) to estimate the daily effective reproductive number (*R*_t_) in each district, using intrinsic incubation period and generation interval distributions from the literature [4–6]. Districts with less than 12 cumulative cases in the study period were excluded from the analysis, and we discarded *R*_t_ estimates obtained before 12 cumulative cases were reported [7]. We computed 95% confidence intervals (95% CI) for each *R*_t_ using an overlapping block bootstrap method and identified periods of local community transmission if the lower bound of the 95% CI was *R*_t_ > 1 for two subsequent weeks or more. We used the estimateR package (R software) to develop the analysis.

**Fig 1:**
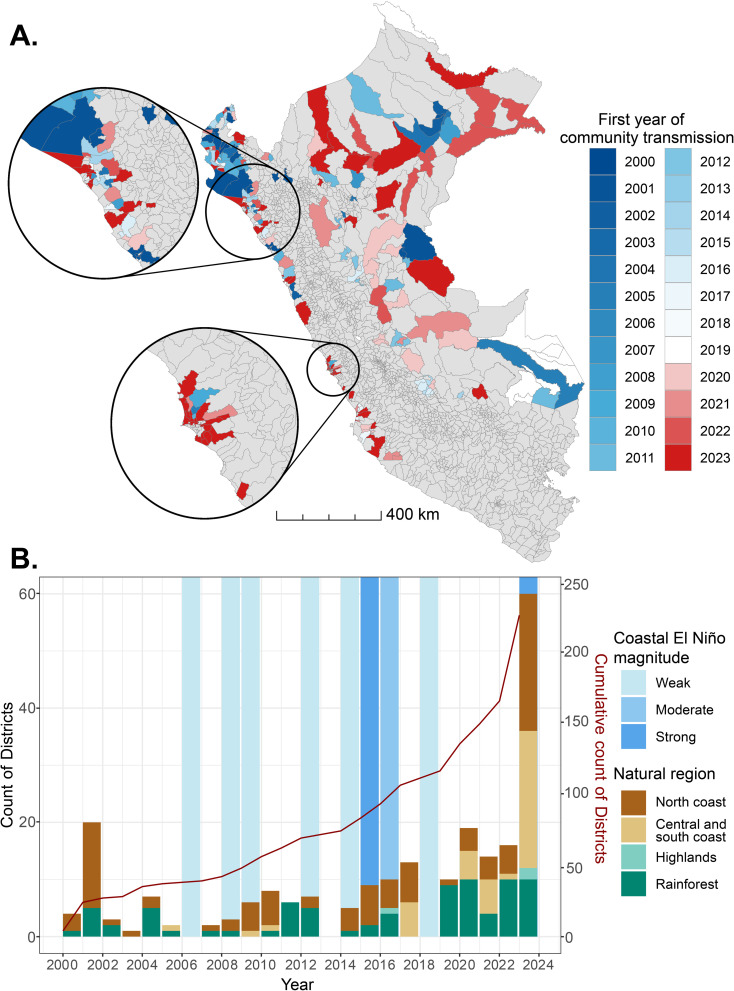
(A) Map of Peru showing the first year of local community dengue transmission in each district, defined as *R*_t_ significantly above 1 for at least 14 consecutive days (2000–2023). Color break in 2019 is due to the Pan American Health Organization (PAHO) declaration of a dengue epidemic in the Americas during that year. (B) Number of districts with local community transmission of dengue for the first time by year and natural region (pre-2000 to 2023). The inset shows the geographic distribution of natural regions in Peru. Light blue shading indicates years with Coastal El Niño events by magnitude. Coastal El Niño: Localized to the coastal areas of Peru and Ecuador, characterized by coastal sea surface warming, has more localized weather impacts, and can occur independently of El Niño-Southern Oscillation (ENSO). ENSO: Large-scale, affects central and eastern Pacific, includes both El Niño and La Niña phases, and has global climate impacts. Maps were produced using R v.4.1 (R Development Core Team, R Foundation for Statistical Computing, Australia) based on public geographic data extracted from INEI, Peru contributors (https://estadist.inei.gob.pe/map) under Open Data Commons Open Database License (ODbL) 1.0 (http://openstreetmap.org/copyright).

The result is concerning. In the last 5 years, a total of 119 districts have detected local community dengue transmission for the first time (Fig 1A). Between 2000 and 2020, the rate of increase in new districts was relatively stable, with a mean of 6.8 new districts per year, and most new districts located in the Amazon rainforest region. However, in 2023 alone, there were 60 new districts with dengue transmission. Compared to previous periods, the most significant difference is observed along the coastal region, where the number of new districts with dengue jumped from an average of 5.6 per year to 48 in 2023 (Fig 1B). Overall, three primary geographic areas of dengue expansion are clearly defined in Peru: the Amazon rainforest, the northern coast, and now the central and southern coast. Finally, the mean daily *R*_t_ was 1.4 (standard deviation [SD] = 2.5), while the daily mean of the 95% CI lower and upper bound were 0.08 (SD = 0.6) and 10.6 (SD = 9.5), respectively.

It has been described that dengue transmission optimal conditions in the Amazon rainforest and northern coast were 21–29 °C and >70% humidity [8]. However, rapid urbanization and the rise in frequency and magnitude of meteorological anomalies such as El Niño-Southern Oscillation (ENSO) influence the report of new dengue community transmission geographic areas [9,10]. Peru has extensive experience in the surveillance and controlling of dengue and other vector-borne diseases such as malaria in the Amazon rainforest region. However, this accumulated experience is not uniformly distributed across the country, and areas without previous exposure to dengue are struggling with the sudden surge in cases. In addition, the expansion of the dengue transmission area along the central and southern coast is worrisome due to the increasing susceptible population, especially in Lima, the most populated and urban city in the country (29.2% of the Peruvian population). Highlands have not been affected yet due to altitude and low temperatures; however, climate change could alter these fragile environmental conditions [11]. In addition, it is important to notice some limitations of data. This data was collected as part of passive case detection (i.e., not all cases of dengue are presented to a health facility). Therefore, case notification depends on the completion of the epidemiological form and samples taken by healthcare personnel; however, in resource-limited settings, this scenario is not feasible in 100% of patients.

Our findings are useful to underscore that, for example, ENSO during 2023 could influence dengue rapid expansion to new geographic areas. The observed phenomenon may be attributed to the dissemination of the dengue virus in previously non-endemic regions where the vector was already established. For instance, in Lima, the capital of Peru—historically considered non-endemic for dengue—the presence of the vector was documented in certain districts several years ago, with reports of vector reemergence dating back to the year 2000 [12]. Within this context, the ENSO may facilitate the rapid expansion of dengue, particularly in densely populated urban areas. Therefore, we advocate for the development of new tools to monitor the dissemination of arboviruses, including the large-scale implementation of trap-based mosquito surveillance, sentinel surveillance of syndromic cases, forecasting models and early warning systems, and effective risk communication, including international cooperation. Such efforts are crucial to increasing the precision of preventive and control actions, ultimately saving lives.
